# Burden in primary informal caregivers of children and adolescents with type 1 diabetes: Is it associated with depression, family dysfunction, and glycemic control?

**DOI:** 10.3389/fendo.2022.1089160

**Published:** 2023-01-19

**Authors:** Lourdes Balcázar-Hernández, Hebert Huerta-Martínez, Eulalia Garrido Magaña, Elisa Nishimura-Meguro, Abigail Jiménez Márquez, Aleida Rivera-Hernández

**Affiliations:** ^1^ Endocrinology Department, Hospital de Especialidades, Unidad Médica de Alta Especialidad (UMAE) Centro Médico Nacional Siglo XXI, Mexico City, Mexico; ^2^ Pediatric Endocrinology Department, Hospital de Pediatría, Unidad Médica de Alta Especialidad Centro Médico Nacional Siglo XXI, Mexico City, Mexico

**Keywords:** type 1 diabetes mellitus, caregiver burden, primary informal caregivers, depression, family dysfunction

## Abstract

**Objective:**

The requirement of a chronic treatment and the increase in life expectancy in children with type 1 diabetes (T1D) leads to the possibility of caregiver burden. The aim of our study was to evaluate the burden in primary informal caregivers (PIC) of children and adolescents with type 1 diabetes and its association with depression, family dysfunction, and glycemic control.

**Materials and methods:**

A retrospective study was performed in PIC of children and adolescents with T1D. Zarit Burden Interview Scale (ZBIS) was used to evaluate caregiver burden. Beck Depression Inventory (BDI-II) was used to evaluate depression in PIC, and the Family APGAR questionnaire was used to evaluate the family functionality.

**Results:**

A total of 100 PIC of children and adolescents with T1D were included. Caregiver burden was found in 33% of caregivers. The total score of the Zarit scale was 41 (34–49); 19% had mild caregiver burden, and 14% had severe caregiver burden. According to the BDI-II, 82% had minimal depression, 11% mild depression, 5% moderate depression, and 2% severe depression. Family function was good in 69%; 13% had moderate dysfunction, and 18% had severe dysfunction. A positive correlation between caregiver burden and BDI-II score (r = 0.84; p = 0.001) and the grade of depression (r = 0.87; p = 0.001) was found. A logistic regression model showed that BDI-II score was associated with caregiver burden (OR 1.14; 95% CI 1.061–1.23; p = 0.001). A BDI-II cut off of 9 or more had a sensibility and specificity of 58% and 28%, respectively, for caregiver burden [AUC 0.751 (0.64–0.85); p = 0.001]. A BDI-II score ≥9 was a predictor of caregiver burden (OR 3.4; 95% CI 1.4–8.1; p = 0.008).

**Conclusion:**

Caregiver burden is present in more than one third of the PIC of patients with T1D and is associated with depression. A BDI-II score ≥9 is a predictor of caregiver burden which may be a point to take into account in the integral approach to the patient with T1D and his or her family nucleus.

## 1 Introduction

Type 1 diabetes (T1D) is a chronic autoimmune disease characterized by pancreatic beta cell destruction, leading to hyperglycemia and to a lifelong insulin-dependent state. The genetic susceptibility combined with environmental factors plays a crucial role in T1D pathophysiology (1). T1D is one of the most frequent chronic diseases in childhood and its incidence is increasing worldwide. In Mexico, the incidence of T1D in children and adolescents is fluctuating, with a reported incidence of 3.4 to 2.8 per 100,000 between 2000 and 2018 in subjects under 20 years of age, which represents an important health problem in our population (2).

The advent of insulin has made it possible to extend the life expectancy of patients with T1D; however, this estimate may vary depending on the population studied and the age at diagnosis of T1D (3, 4).

The increase in life expectancy leads to the need for primary informal caregivers (PIC) and, with it, the possibility of caregiver burden.

Informal caregivers are a critical resource for their recipients and an essential component of health care systems. An informal caregiver, often a family member, provides care to someone with whom he or she has a personal relationship, and is usually unpaid (5).

The PIC face psychosocial challenges that include high levels of psychological symptomatology, reduced social connectedness, and caregiver burden (6). Caregiver burden represents the degree to which caregivers perceive that caring for their patient has had an adverse effect on their emotional, social, financial, physical, and spiritual functioning (7).

It has been reported that caregivers with a high level of emotional overload show a worse self-perception of their health and a higher probability of presenting emotional disorders (8). Some research has documented that depression is the most frequent disorder in PIC, along with anxiety, despondency, and discouragement (9, 10). However; most reports have been in caregivers of adult patients.

In Mexico, most of the medical care in the pediatric population corresponds to the family, particularly in children with T1D. Despite this, little is known about the emotional impact of chronic diseases on PIC. In our population, all caregivers of adult patients with chronic pain have been reported to have multiple symptoms of depression (11); however, information about caregiver burden in caregivers of children and adolescents with chronic illness is lacking.

The aim of our study was to evaluate the burden in PIC of children and adolescents with type 1 diabetes and its association with depression, family dysfunction, and glycemic control.

## 2 Materials and methods

### 2.1 Patients

A cross-sectional study was performed in PIC of children and adolescents with T1D enrolled in the clinic of diabetes at a Pediatric Tertiary Care Center in Mexico City between June 2018 and January 2020. Sociodemographic variables of PIC included age, gender, marital status, relationship to the patient, schooling, socioeconomic level, and occupation. PIC with a previous diagnosis of psychiatric disorder were excluded, as well as those in which complete information was not obtained.

The glycemic control was determinate by glycated hemoglobin (HbA1c). Patients with HbA1c 7.5% or below were considered under control and those with HbA1c >7.5% were considered with decontrolled diabetes (12). HbA1c and time of evolution of T1D was evaluated at the time of caregiver burden evaluation.

The Zarit Burden Interview Scale (ZBIS) was used to evaluate caregiver burden. ZBIS is a validated questionnaire in our population (internal consistency: Cronbach alpha coefficient of 0.84). A score ≤46 excluded caregiver burden, a score between 47 and 55 indicated low caregiver burden, and a score ≥56 indicated intense caregiver burden (13).

The Beck Depression Inventory (BDI-II) was used to evaluate depression in PIC. This inventory has been validated in our population (internal consistency: Cronbach alpha coefficient of 0.87) (29). According to the recommendations in our population, depression was categorized in: minimal depression (score form 0–9), mild (score from 10 to 16), moderate (score from 17 to 29 items puntos), and severe (score from 30 to 63) (14, 15).

The Family APGAR questionnaire was used to evaluate family functionality. The five functional components of the Family APGAR were: Adaptability, Partnership, Growth, Affection, and Resolve. Family function can be categorized as “good” (score from 7 to 10), “moderate dysfunction” (score from 4 to 6), or “severe dysfunction” (score from 0 to 3) (16).

The ZBIS, BDI-II, and Family APGAR questionnaire were performed on those PIC who agreed to participate in the study by the same expert investigator during the doctor’s appointment of patients with T1D. These questionnaires are not routinely administered at the medical visit. Demographic, clinical, and biochemical data were obtained during the routine appointment and from the electronic medical records by the same investigator.

### 2.2 Statistical analysis

The continuous variables were described as median and interquartile range (IQR). For the categorical variables, proportions were used (expected frequency, prevalence). Two-sample *t*-test or Mann–Whitney *U* test were used to compare continuous variables according to the distribution, and for the categorical variables, the χ^2^ test was used. Correlations of quantitative variables were performed using the Spearman’s rank test. Multivariable logistic regression adjusted by BDI-II score and grade of depression was used to identify risk factors for caregiver burden. An ROC curve was used to estimate the sensibility and specificity of the BDI-II cutoff point for predicting caregiver burden. The area under the ROC curve (AUC), positive predictive value (PPV), negative predictive value (NPV), and likelihood ratios (LR+ and LR-) were used to the measure of test performance. All statistical tests were two-tailed; p < 0.05 was considered statistically significant. We used IBM SPSS Statistics V25.0 (IBM SPSS ^®^, EEUU) and STATA V14 (StataCorp ^®^, EEUU) as statistical software.

## 3 Results

### 3.1 Baseline characteristics of patients with type 1 diabetes and their caregivers

Of a total of 110 potential PIC of children and adolescents with T1D, 100 were included. Of the excluded PIC, eight had a diagnosis of anxiety and/or depression and in two the information obtained was incomplete.

### 3.2 Patients

The age at diagnosis was 11 years (7–14); 89% were women. The evolution time of T1D since the diagnosis was 2.2 (1–4.7) years, and 18% had an adequate control of diabetes in the last 6 months (**Table 1**).

**Table 1:** Characteristics of children and adolescents with type 1 and differences according to the presence of burden in their primary informal caregivers.

Caregiver burden					
Variable	Total (n = 100)	With n = 33	Without n = 67	p Value	95% Confidence interval
Age (years)	11 (7–14)	10.4 (8.1–12.8)	10.5 (7.9–12.5)	0.86	0.82–0.91
Gender; % (n) Female Male	48 (48) 52 (52)	48.5 (16) 51.5 (17)	47.8 (32) 52.2 (35)	0.94	0.89–0.97
Time of diabetes evolution (years)	2.2 (1–4.7)	2 (1–4.5)	3 (1–4)	0.29	0.17–0.32
HbA1c (%)	8.5 (7.8–9.7)	8.4 (7.5–9.5)	9 (8.2–10.6)	0.08	0.07–0.93
Control of diabetes; % (n) HbA1c <7.5% HbA1c >7.5%	18 (18) 82 (82)	12.1 (4) 87.9 (29)	22.4 (15) 77.6 (52)	0.22	0.19–0.3

Quantitative variables reported at median and interquartile range.

### 3.3 Caregivers

The age at diagnosis was 40 years (36–46); 89% were women. Of the caregivers, 80% were represented by the patient’s mother, 55% had a paid employment in their role as primary caregiver, and 58% attended the “Diabetes Boot Camp” program. The characteristics of caregivers are summarized in **Table 2**. Caregiver burden was found in 33% (95% CI 0.23–0.4).

**Table 2:** Characteristics of primary informal caregivers and differences according to the presence of caregiver burden.

Caregiver burden					
Variable	Total (n = 100)	With n = 33	Without n = 67	p Value	95% Confidence interval
Age (years)	40 (36–46)	40 (35–45)	43 (39–46)	0.08	0.07–0.088
Gender; % (n) Female Male	89 (89) 11 (11)	87.9 (29) 12.2 (4)	89.6 (60) 10.4 (7)	0.80	0.77–0.88
Relationship; % (n) Mother Father Grandmother	80 (80) 11 (11) 9 (9)	81.8 (27) 12.1 (4) 6.1 (2)	79.1 (53) 10.4 (7) 10.4 (7)	0.69	0.62–0.71
Education; % (n) Primary Secondary High school completed Bachelor’s degree	16 (16) 29 (29) 41 (41) 14 (14)	9.1 (3) 24.2 (8) 45.5 (15) 21.2 (7)	19.4 (13) 31.3 (21) 38.8 (26) 10.4 (7)	0.06	0.056–0.8
Socioeconomic Status; % (n) Low Medium High	31 (31) 68 (68) 1 (1)	27.3 (9) 69.7 (23) 3 (1)	32.8 (22) 67.2 (45) 0	0.44	0.41–0.51
Occupation; % (n) Housewife Merchant General Employee Professional Unemployed	43 (43) 4 (4) 45 (45) 6 (6) 2 (2)	42.4 (14) 6.1 (2) 48.5 (16) 3 (1) 0	43.3 (29) 3 (2) 43.3 (29) 7,5 (5) 3 (2)	0.67	0.65–0.69
Marital Status; % (n) Single Married Divorced Unmarried	19 (19) 65 (65) 4 (4) 12 (12)	12.1 (4) 69.7 (23) 6.1 (2) 12.1 (4)	22.4 (15) 62.7 (42) 3 (2) 11.9 (8)	0.30	0.23–0.53
Diabetes Camp; % (n)	58 (58)	66.7 (22)	53.7 (36)	0.22	0.19–0.39
Zarit score	41 (34–49)	37 (30–41)	53 (50–59)	0.001	0.00–0.00
Beck Depression Inventory score	6 (4–11)	12.5 (6.5–16.5)	4 (2–7)	0.002	0.00–0.00
Family APGAR score	8 (5–10)	8 (5–10)	7 (5–9)	0.21	0.18–0.33

Quantitative variables reported at median and interquartile range.

### 3.4 Caregiver burden, depression, and family function in caregivers

Caregiver burden was found in 33% of caregivers (95% CI 0.23–0.4). The total score of Zarit scale was 41 (34–49); 19% had mild caregiver burden and 14% had severe caregiver burden. Sixty-six percent of caregivers with burden corresponded to patients older than 10 years. The items with the highest scores were: fear of the future, feeling that the patient is dependent, and feeling of having to do more and better. The most affected subscales were interpersonal functioning and self-care activities.

According to the BDI-II, 82% had minimal depression, 11% mild depression, 5% moderate depression, and 2% severe depression. The total BDI-II score was 7 (4–11).

The APGAR score was 8 (5–10). Family function was good in 69%; 13% had moderate dysfunction and 18% had severe dysfunction.

The most affected subscales in PIC with burden were self-care according to ZBIS (78.7%), somatic-affective area (63.6%) according to BDI-II, and partnership (12%) according to APGAR questionnaire (**Table 3**).

**Table 3:** Sub-scales of the Zarit Burden Interview Scale, Beck Depression Inventory and Family APGAR questionnaire affected in burdened caregivers (n = 33).

Instrument	Sub-scales
Zarit Burden Interview Scale; % (n)	Impact on self-care: 78.7 (26) Alterations in interpersonal relationships: 45.4 (15) Self-efficacy expectations: 51.5 (17)
Beck Depression Inventory; % (n)	Somatic-affective area: 63.6 (21) Cognitive area: 42.4 (14)
Family APGAR questionnaire; % (n)	Adaptation: 3 (1) Partnership: 12 (4) Growth: 9 (3) Affection: 3 (1) Resolve: 6 (2)

### 3.5 Caregivers and patients’ differences according to the presence of caregiver burden

Caregivers with burden were younger than those without burden [40 (35–45) *vs*. 43 (39–46); p = 0.08]. On the other hand, BDI-II score was higher in caregivers with burden compared to those without burden [12.5 (6.5–16.5) *vs*. 4 (2–7); p = 0.002]. There were no differences in gender, relationship, scholarliness, occupation, marital status, family APGAR score, and patient clinical parameters as age, gender, and diabetes control (**Tables 1**, **2**). There were no differences in age [11 (7.5–14) *vs*. 10.5 (5.5–13) years; p = 0.67, 95% CI 0.56–0.8], time of diabetes evolution [3 (2–5.7) *vs*. 3.2 (1.5–5) years; p = 0.79, 95% CI 0.63–0.85], HbA1c [8.3 (7.9–9.4) *vs*. 9.8 (7.9–11.25) %; p = 0.23, 95% CI 0.19–0.39], and control of diabetes (p = 0.90, 95% CI 0.85–0.96) of patients comparing PIC with mild and severe burden.

### 3.5 Factors associated with caregiver burden

A strong positive correlation between caregiver burden (ZBIS) and BDI-II score (r=0.84; p = 0.001) and the grade of depression (r = 0.87; p = 0.001) was found. There were not association between caregiver burden, sociodemographic variables of caregivers, family APGAR score and clinical characteristics of patients (**Table 4**). A logistic regression model adjusted by BDI-II score and grade of depression showed that BDI-II score was associated with caregiver burden (OR 1.14; 95% CI 1.061–1.23; p = 0.001), without evidence of association with the grade of depression (OR 1.06; 95% CI 0.45–33.7; p = 0.09).

**Table 4:** Associations between caregiver burden and sociodemographic variables of caregivers, the Beck Depression Inventory score, the family APGAR score, and clinical characteristics of patients.

	r_s_ Value	95% Confidence interval	p Value
Caregivers’ characteristics			
Age	0.17	0.02–0.35	0.83
Gender	0.25	0.05–0.42	0.80
Relationship	0.03	-0.16 to 0.22	0.69
Education	0.19	-0.006 to 0.37	0.51
Socioeconomic status	0.76	0.66–0.83	0.45
Occupation	0.42	0.24–0.56	0.67
Beck Depression Inventory score	0.84	0.77–0.88	0.001
Grade of depression	0.87	0.81–0.91	0.001
Family APGAR score	0.12	-0.07 to 0.30	0.21
Patients’ characteristics			
Age	0.18	0.01–0.36	0.86
Gender	0.07	-0.12 to 0.26	0.94
Type 1 diabetes evolution	0.10	-0.09 to 0.29	0.29
HbA1c	0.17	-0.02 to 0.35	0.08
Diabetes control	0.11	-0.08 to 0.29	0.25

A BDI-II cut off of 9 or more had a sensibility and specificity of 58 and 72%, respectively, for caregiver burden [AUC 0.751 (95% CI 0.64–0.85); p = 0.0001], with a PPV of 50%, a NPV of 77%, a LR+ of 2.07, and a LR- of 0.58 (post odds +1.02), with a post-test probability of caregiver burden of 50%. A BDI-II score ≥9 was a predictor of caregiver burden (OR 3.4; 95% CI 1.4–8.1; p = 0.008).

## 4 Discussion

In this study, we show that more than one third of the PIC of patients with T1D have caregiver burden according to the Zarit scale. In PIC the main areas affected are interpersonal functioning and self-care activities. Likewise, most patients have depression despite a good family function.

Supporting children with T1D is complex and requires a great deal of effort on the part of the caregiver. There are five inter-related support needs in children with T1D: children need time to adjust to the diagnosis, need supportive relationships, need an opportunity for meaningful participation and appropriate protection, need to engage and explore, and need to feel supported, but not different (17).

The complexity of these needs represents a challenge for the caregiver of the child and adolescent with T1D, which can lead to emotional disturbances such as burden, anxiety, and stress. Mothers of children with newly diagnosed diabetes experience negative consequences in their occupational situation, and this inequality can have long-term negative consequences for their mental health and future economic situation (18).

A recent study reported that 68.9% of mothers of children and adolescents with T1D present moderate to severe burden (19). In our population, the frequency was lower, probably related to the fact that caregivers have emotional tools that allow them to cope with T1D in a better way compared to other populations; however, it is important to highlight that this study was conducted in a tertiary hospital, which implies that patient care may be more supervised, requiring replication of this study design in other populations, taking our finding as a reference.

In children with chronic disease, children’s number of medicines and injections, a diagnosis of attention-deficit/hyperactivity disorder in addition to the primary medical condition, frequent primary care provider and emergency room visits, and lower child self-efficacy were predictors of increased caregiver burden (20). Some factors have been associated with caregiver burden in patients with T1D. An inverse correlation between burden and physical health, social relationships, psychological health, environment, and quality of life has been evidenced (19).

Kobos et al. reported that the level of burden in caregivers of children with T1D correlated with the child’s age, the professional status and level of education of the parents, the number of glycemic tests at nighttime, the frequency of hyperglycemic episodes, and the number of hospitalizations (21); however, in our study there was no significant correlation with any of these variables; likewise, we did not observe a higher number of hospitalizations or a lower educational level of the PIC.

In our study, we evidenced an association of caregiver burden and BDI-II score. These data have not been previously published in the pediatric population with D1T, providing a novel finding and a turning point for future studies. We showed that a BDI-II score ≥9 was a predictor of caregiver burden; however, this data should be taken with caution due to low sensibility and specificity despite an acceptable AUC, low PPV, and small increase in the probability of caregiver burden according to the likelihood ratio.

The caregiver’s burden has consequences in other aspects of his or her life. Caregiver burden has been negatively associated with parents’ quality of life. Resilience serves as a moderator between caregiver burden and mental health, and it is positively associated with life quality. The benefit of high resilience for better mental health in parents with caregiver burden has been proposed (22).

In families with very young children with T1D, parental perceptions of the burden of managing diabetes are common and could be mitigated by tailored education programs that increase parent knowledge, bolster parents’ confidence in themselves, and increase trust in their secondary caregivers to manage diabetes. Reduced parental burden and increased caregiver knowledge may positively impact a child’s glycemic control, as well as improve parent and child quality of life (23)

Among the strengths of our study is the availability of information in a relatively acceptable sample size given the rarity of the disease, as well as the application of three instruments adequately validated in the studied population, which assesses caregiver burden, depression, and family functionality in a small studied population of primary caregivers. In addition, we found an association of caregiver burden with BDI-II scores and, indirectly, with depression. Limitations include its cross-sectional nature, the low sensitivity and specificity of the BDI-II score found in the ROC curve to predict caregiver burden, the difficulty in identifying the direction between depression and caregiver burden, as well as possible selection biases, especially because it was conducted in a tertiary hospital.

Our study denotes the importance of the routine application of instruments aimed at the diagnosis of burden or depression in ICP, and with it, the application of timely prevention or treatment strategies that could influence the long-term prognosis of patients with T1D.

## 5 Conclusion

Caregiver burden is present in more than one-third of the PIC of patients with T1D and is associated with depression. A BDI-II score ≥9 is a predictor of caregiver burden which may be a point to take into account in the integral approach to the patient with T1D and his or her family nucleus.

## Data availability statement

The raw data supporting the conclusions of this article will be made available by the authors, without undue reservation.

## Ethics statement

The project was reviewed and approved by the ethics and research committee of the Hospital de Pediatría Dr. Silvestre Frenk Freund, Centro Médico Nacional Siglo XXI of the Instituto Mexicano del Seguro Social. Written informed consent to participate in this study was provided by the participants’ legal guardian/next of kin.

## Author contributions

All authors contributed substantially to the conception and design of the study, the collection and analysis of data, and the review and editing of the draft. All authors contributed to the article and approved the submitted version.
